# Safety and effectiveness of laparoscopic Y-en-Roux gastric bypass surgery in obese elderly patients^1^

**DOI:** 10.1590/s0102-865020200060000006

**Published:** 2020-07-06

**Authors:** Marcelo Protásio dos Santos, José Eduardo Gonçalves, André Akira Ramos Takahashi, Bruno Barros Britto, Fernando Bray Beraldo, Jaques Waisberg, Luciana Kase Tanno

**Affiliations:** I MD, MsC. Department of Surgery, Hospital do Servidor Público Estadual (IAMSPE), Sao Paulo-SP, Brazil. Design of the study, interpretation of data, manuscript preparation.; II PhD, Department of Surgery, IAMSPE, Sao Paulo-SP, Brazil. Interpretation of data.; III MD, Department of Surgery, Faculdade de Medicina do ABC, Santo Andre-SP, Brazil. Manuscript preparation, critical revision.; IV MD, Department of Surgery, Faculdade de Medicina Tiradentes, Aracaju-SE, Brazil. Interpretation of data, manuscript preparation.; V MD, Department of Surgery, IAMSPE, Sao Paulo-SP, Brazil. Acquisition and interpretation of data.; VI PhD, Department of Surgery, IAMSPE, Sao Paulo-SP, and; Department of Surgery, Faculdade de Medicina do ABC, Santo Andre-SP, Brazil. Design of the study, interpretation of data, manuscript preparation, critical revision.; VII PhD, Division of Allergy, Department of Pulmonology, Hôpital Arnaud de Villeneuve, University Hospital of Montpellier, Paris, France. Analysis of data, manuscript preparation, critical revision.

**Keywords:** Anastomosis, Roux-en-Y, Bariatric Surgery, Aged

## Abstract

**Purpose:**

To analyze, in aged obese patients, the weight loss, comorbidity control, and safety postoperative complications of bariatric surgery by Roux-en-Y gastric bypass technique.

**Methods:**

Twenty-seven patients who underwent laparoscopic weight-reducing gastroplasty with Roux-en-Y gastric bypass to treat obesity were included. All patients were ≥ 60 years old at the time of surgery. The Wilcoxon test was used for statistical analysis, and a p-value ≤0.05it was considered significant.

**Results:**

Ten (90.9%) patients with dyslipidemia were cured (p < 0.001). Nine (81.8%) patients with type 2 diabetes mellitus had total improvement and 2 (18.2%) had partial improvement (p = 0.003). In 23 patients with systemic arterial hypertension, 9 (39.1%) achieved total improvement and 14 (60.9%) partial improvement (p = 0.140). Five (71.4%) patients with obstructive sleep apnea syndrome were cured (p = <0.001). For other comorbidities, no partial improvement or cure was shown.

**Conclusions:**

Roux-en-Y gastric bypass surgery in obese elderly patients can be performed safely and with low morbidity and mortality rates. The benefits of weight loss and reduced comorbidities are promising and like those of the younger population.

## Introduction

Obesity, given the significant associated morbidity and mortality rates, is considered a worldwide epidemic with high costs for society and health systems^1^. In 2014, the World Health Organization (WHO) estimated that approximately 1.9 billion adults worldwide were overweight and at least 600 million were obese^2^. The major medical concern regarding obesity is the large number of associated diseases, especially cardiovascular disease, diabetes, and malignant neoplasms^3^. Several long-term epidemiological studies have been able to demonstrate this strong association, as well as evidence of reduced quality of life and increased mortality in these patients^4-6^.

Although clinical treatment shows good results, the Roux-in-Y gastric bypass (RYGB) procedure is considered the gold standard for weight loss, and it has been the most widely performed bariatric procedure worldwide. Nevertheless, nowadays, vertical gastrectomy is considered the most frequently performed operation^7^. The gastric pouch has considerably smaller volume (60 ml), which facilitates the lower consumption of food and calories. In addition, there is less assimilation of calories and nutrients.

The premature existence of ingested foods that pass into the distal small intestine may stimulate secretion of substances such as glucagon-1 peptide, leading to improved insulin sensitivity, increased insulin production and decreased resistance to insulin. This improvement in insulin sensitivity has proven to be an independent factor of weight loss^8^. Due to duodenum bypass, RYGB has the potential to cause vitamin deficiencies, as well as deficiencies in minerals such as iron, copper, selenium, zinc, calcium and phosphorus^9^.

The elderly population represents a growing share of the world’s population. It is estimated that 8% of people worldwide are over 75, and by 2025, this percentage is estimated to reach 10%^10^. The population over 60 years old already corresponds to 23% of all surgical procedures performed in the world. However, the number of elderly people undergoing surgery is increasing more than the elderly in the general population^11^. A significant portion of elderly patients present higher risks of morbidity and mortality in emergency and even elective procedures when compared to patients of other age groups^12^.

The prevalence of obesity among the elderly is increasing, and it is estimated in 17.9% on 65 years old^13^. Increased waist circumference was present in 47.6% of people^13^. The sarcopenic obesity, defined as the coexistence of sarcopenia and obesity, significantly affects the exercise of daily activities, affecting the quality of life and even the number of hospitalizations^14^. In addition, another problem of immobility and high body mass index in the elderly is the increased incidence of pressure ulcers. A retrospective cohort study found that hospitalized and intensive care patients with grade III obesity (BMI ≥ 40kg/m^2^) had significantly more pressure ulcers than other patients, as well as a higher mortality rate^15^.

As a result of this scenario, surgical procedures to treat obesity in the elderly are increasing and represent about 10% of the total bariatric surgeries performed in academic centers^16,17^. The majority of the studies advocates in favor of the safety and efficiency of bariatric surgery; however, a deeper knowledge is needed to examine the outcome in elderly patients. The perioperative morbidity and mortality in elderly patients are the central concern for bariatric surgery in this population. This issue associated with the lack of studies, contributes for medical care centers to deny bariatric surgery coverage to patients over the age of 60 years^16^.

The approach to combating obesity in the geriatric population still lacks solid information and well-established methods because it is a recent research area and many doubts are still observed in this practice^18^. Thus, due to the increase in obese elderly and the lack of consensus on the efficacy and safety of bariatric surgery for these patients, we consider it important to analyze the results of gastric bypass surgery in ≥ 60 year-old obese patients.

The aim of the present study was to analyze the results of comorbidity control before and after 2 years of laparoscopic RYGB in ≥ 60 year-old obese patients and to identify possible postoperative complications of this procedure in this population.

## Methods

The research protocol for this study was approved by the Research Ethics Committee of the institution.

This retrospective study was performed with adult patients of both genders who underwent laparoscopic RYGB. All surgeries were performed using Roux-en-Y gastroplasty with a 60cm long biliopancreatic loop; the length of the alimentary loop was 120cm and a 3-4cm gastric pouch was created. One of the patients also received a non-adjustable silicone ring around the gastric pouch. All surgical procedures were performed at the Department of Surgery (Gastrointestinal Surgery Division) of the Hospital do Servidor Público Estadual. Clinical and laboratory data were obtained from medical records of patients that underwent an operation between January 2011 and January 2014.

Inclusion criteria for patients in this study were age ≥ 60 years, body mass index (BMI) ≥ 40 kg/m^2^ regardless of the presence or absence of comorbidities, BMI ≥ 35 kg/m^2^ associated with one or more comorbidities, and acceptance of the consent form after all the benefits and risks offered by the operation were clarified. The exclusion criteria adopted were patients submitted to other modalities of bariatric operations or who underwent the same surgical procedure with an open approach.

We studied 25 (92.6%) women and 2 (7.4%) men, regardless of ethnicity. The average age of the patients was 63.1 ± 3.6 years (60 to 75 years old).

All patients had grade II or III obesity according to WHO criteria, whether or not it was associated with one or more of the following comorbidities: arthropathy, asthma, depression, dyslipidemia (DLP), type 2 diabetes mellitus (T2DM), gastroesophageal reflux disease (GERD), systemic arterial hypertension (SAH), hypothyroidism, and obstructive sleep apnea syndrome (OSAS)^2^.

The absence of the need for medication for postoperative comorbidity control was considered a cure, and the need for medication at a lower dose than the preoperative dose was considered partial comorbidity control.

The quantitative variables analyzed were age, length of hospital stay, maximum patient weight, preoperative weight, weight after 24 months of operation, percentage of excess weight loss (%EWL), maximum patient BMI, preoperative BMI, BMI 24 months after surgery, number of comorbidities, number of preoperative medications, and medications used to control comorbidities 24 months after surgery.

Statistical analysis used mean with standard deviation, median and percentages. The nonparametric Wilcoxon test and confidence intervals were employed. The significance level adopted was 0.05 (5%).

## Results

The average length of hospital stay was 5.67 ± 1.73 days (4 to 12 days). The average maximum weight was 116.6 kg ± 18.2 (93 kg to 180 kg) and the average weight after 24 months of follow-up was 73.1 kg ± 11.7 (50 kg to 100 kg). The mean %EWL was 74.6% ± 22.2 (31.8% to 135.6%) (Table 1).

**Table 1:** Description of quantitative variables of elderly patients undergoing laparoscopic Roux-en-Y gastric bypass.

Variables	Mean (Min-Max)	Median	SD
Age (years)	62 (60-75)	62	3.6
Days of hospitalization	5.67 (4-12)	5	1.73
Maximum weight (kg)	116.6 (93-180)	110	18.2
Preoperative weight (kg)	104.3 (83-170)	103	16.6
Weight after 24 months (kg)	73.1 (50-100)	70	11.7
%EWL after 24 months	74.6% (31.8-135.6)	73.2	22.2
Maximum BMI	46.6 (38-60)	46	5.5
Preoperative BMI	42.0 (33.8-54)	43.0	4.3
BMI after 24 months	28.3 (22-33)	29	3.3
No. of comorbidities	2 (0-5)	2	1.33
No. of preoperative medications	6 (0-15)	6	3.93
No. of medications after 24 months	3 (0-10)	3	1.84

Min. = minimum; Max. = maximum; SD = standard deviation; %EWL= % of excess weight loss; BMI = body mass index.

The average of maximum BMI was 46.6 kg/m^2^ ± 5.5 (38 kg/m^2^ to 60 kg/m^2^) and the mean preoperative BMI was 42.0 kg/m^2^ ± 4.3 (33.8 kg/m^2^ to 54 kg/m^2^). After 24 months of evaluation, the mean BMI was 28.3 kg/m^2^ ± 3.3 (22 kg/m^2^ to 33 kg/m^2^) (Table 1).

Before surgery, the patients used five medications per day on average to control obesity-associated comorbidities (T2DM, SAH, and DLP). After 24 months, there was a reduction to three medications on average per day per patient (Table 1).

The average number of obesity-associated comorbidities per patient was 2. Of the 27 patients, 25 (92.6%) had one or more obesity-related diseases. In this group of patients, the distribution of comorbidities is shown in Table 2.

**Table 2:** Distribution of comorbidities in elderly patients before and after undergoing laparoscopic Roux-en-Y gastric bypass.

Comorbidities	Preoperative period N (%)	Postoperative period N (%)	P-value
Arthropathy	2 (7.4%)	1 (3.7%)	0.2 (N.S.)
Asthma	2 (7.4%)	1 (3.7%)	0.2 (N.S.)
Depression	1 (3.7%)	0	0.1 (N.S.)
Dyslipidemia (DLP)	11 (40.7%)	1 (3.7%)	<0.001*
Type 2 diabetes mellitus (T2DM)	11 (40.7%)	9 (33.3%)	0.003*
Gastroesophageal reflux disease (GERD)	1 (3.7%)	0	0.1 (N.S.)
Systemic arterial hypertension (SAH)	23 (85.2%)	9 (33.3%)	0.14 (N.S.)
Hypothyroidism	5 (18.5%)	2 (7.4%)	0.09 (N.S.)
Obstructive sleep apnea syndrome (OSAS)	7 (25.9%)	2 (7.4%)	<0.001*

Wilcoxon test; * = Significant; N.S. = Not significant.

Regarding comorbidities, of the 11 patients who presented with DLP, 10 (90.9%) were cured (p < 0.001). Of the 11 patients with T2DM, 9 (81.8%) had total improvement and 2 (18.2%) had partial improvement (p = 0.003). Concerning the 23 patients with SAH, 9 (39.1%) achieved total improvement (p = 0.140) and 14 (60.9%) achieved partial improvement. Regarding OSAS, of the 7 (28.0%) patients who presented this comorbidity before the operation, in 2 (8.0%), OSAS remained after 24 months (p = <0.001). For other comorbidities, no partial improvement or cure was shown.

Regarding the operative complications, it was observed that among the 27 patients in the study, 1 (3.7%) had incisional hernia, 1 (3.7%) developed surgical wound infection, and 5 (18.5%) had other postoperative complications: 2 hypertensive crises, 2 bronchospasms and 1 atrial fibrillation with high ventricular response. At long-term follow-up, 1 patient presented solids dysphagia after 2 years with resolution after ring removal surgery. There were no deaths in this series.

## Discussion

The safety and efficacy of weight loss surgical procedures in the elderly is still controversial. Sugerman *et al*.^19^ evaluated 850 elderly patients undergoing RYGB and indicated an operative mortality rate of 0.6% and morbidity of 5.6%. The authors concluded that gastric bypass is a safe procedure in the elderly population due to low morbidity and mortality rates^20^. In the present study, there was no mortality, while the morbidity index was 29.6%. Yoon *et al*.^21^ compared the outcomes of RYGB and vertical gastrectomy among populations < 60 years and ≥ 60 years of age and found no significant differences between the two populations regarding unfavorable outcomes. Postoperative complication rates among the population < 60 years and ≥ 60 years were, respectively, 2.5% and 5%. Mean operative time was slightly longer in patients ≥ 60 years (210 minutes *vs*. 229 minutes) and readmissions at 30 days occurred more frequently in the ≥ 60 year-old group (2.5% *vs*. 12.5%). However, the reasons for hospital readmissions in any of the groups were not detailed in this study^21^. In the present series, no hospital readmissions were recorded within 30 days after the surgical procedure.

Santo *et al*.^22^ evaluated 538 patients undergoing RYGB and found that the most frequent complications were surgical site infection (3.2%), bleeding (2.6%), and intestinal obstruction (1.1%). The main clinical complication was pulmonary thromboembolism, found in 3 (0.55%) patients. The total mortality rate of the study was 0.55% and was related to the presence of pulmonary thromboembolism and age > 65 years. Trieu *et al*.^23^ noted that the most frequent early complications in patients > 60 years of age were anastomotic dehiscence (2.2%), bleeding (1.1%), pulmonary thromboembolism (1.1%), pneumonia (1.1%), and atrial fibrillation (1.1%). Late complications included gastrojejunal anastomosis stenosis (8.6%), peptic ulcers (3.2%), intestinal obstruction (1.1%), internal hernias (1.1%), and abdominal wall hernias (1.1%). The authors did not report deaths in their clinical registries. Pajecki *et al*.^24^ in a study with morbidity and mortality results of elderly patients undergoing RYGB, had similar follow-ups to the previously mentioned studies, except that the mortality rate was 4.3% (2 patients), one death being caused by pulmonary thromboembolism and the other due to sepsis resulting from surgical wound infection. The researchers concluded that pulmonary thromboembolism and age above 65 years are predictors of mortality.

Older age is a predictor of increased length of hospital stay, although there is no significant increase in mortality. This has been shown in studies that found individuals over 65 to be most likely to require prolonged hospitalization^25,26^. Robert *et al*.^27^ revealed that the length of hospital stay after gastric bypass was similar in age groups < 40 years, 40-55 years, and > 60 years. Following the present clinical registry, there was an average length of hospital stay of 5.67 days, with a minimum of 4 days and a maximum of 12 days. It is important to mention that during the period of the operations, the standardized hospitalization time was 4 days for patients undergoing gastroplasty, regardless of age.

Regarding %EWL as a result of RYGB, studies point to the effectiveness of this technique in the elderly. Sugerman *et al*.^19^, in a study of 80 patients aged between 60 and 66 years, weighing between 110 and 154 kg and with BMI between 42 and 56 kg/m^2^, followed these patients for 5 years. After 1 year, patients had weight loss between 27 and 49 kg and a reduction in BMI to 27 to 43 kg/m^2^. Pajecki *et al*.^24^ found that %EWL was 71.8%, similar to the results of the present study, in which an average %EWL of 74.6% was obtained after 24 months.

Zaveri *et al*.^28^ analyzed 57 patients aged ≥ 70 years undergoing various modalities of bariatric surgery and followed them for 18 months. The authors compared the results of laparoscopic gastric band (24 patients), RYGB (14 patients), and duodenal switch (15 patients). The results obtained of %EWL of BMI lost at the end of follow-up were 37.2% for gastric band, 88.4% for gastric bypass and 100.6% for duodenal switch^28^. Huang *et al*.^29^ observed that after performing RYGB, %EWL was 65% in elderly patients. Abbas *et al*.^30^ obtained similar results because the mean %EWL after 12 months of RYGB in patients with a mean age of 63.4 years was 65.2%.

Gray *et al*.^31^ in a retrospective study, analyzed weight loss in 69 elderly patients who underwent laparoscopic RYGB and obtained a percentage of total weight reduction of 23.9 ± 9.3%. These authors found that the percentage of weight loss after 3 years of gastric bypass in the elderly was lower than in young adults (27.5 ± 8.6% *vs*. 31.8 ± 12.1%), as well as a reduction in BMI (12.7 ± 4.9 *vs*. 15.2 ± 6.2). In the present clinical registry, a reduction in the mean BMI after 24 months of 13.7 kg/m^2^ (from 42 kg/m^2^ to 28.3 kg/m^2^) was observed, which was similar to the results of the study by Thereaux *et al*.^32^Although considered effective in this population, results of RYGB in the elderly still diverge in terms of the effectiveness of weight loss.

Regarding the improvement or resolution of comorbidities, Zaveri *et al*.^28^ showed that in 83.3% of patients over 70 years undergoing RYGB, there was a resolution of T2DM in 60% of cases, of OSAS in 65% of cases, of SAH in 50% of cases and of GERD in 50% of cases.

In the present study, 81.8% of patients had achieved T2DM improvement without the need for medication to control the glycemic level. Regarding hypertension, we obtained a resolution percentage of 39.1% and a reduction of 84.6% of OSAS. Pajecki *et al*.^24^ studied patients aged ≥ 60 years who underwent RYGB and were followed for 39 months. The authors observed an improvement in comorbidities such as T2DM, SAH, DLP, and GERD^31^. In 23% of patients there was partial improvement of T2DM, while in 77%, total control of these comorbidities was achieved. Regarding SAH, in 26% of the cases, there was control with medication reduction, while in 30% the control was obtained without medication. The mean glycated hemoglobin of these patients decreased from 6.73% to 5.7%. In the lipid profile, there was a significant increase in HDL, a slight reduction in LDL, and a strong reduction in triglycerides. Ramirez *et al*.^33^ showed reduced use of antidiabetic drugs in 40% of patients, complete resolution of T2DM in 33% of cases, and decreased use of medications for SAH (56%), DLP (54%), and degenerative knee disease (50%). In the present study, there was a reduction of 47.9% in the use of medications to control comorbidities after 24 months (from 5.33 to 3.07 medications) and an improvement in DLP in 90.9% of patients.

The National Hospital Discharge Survey and the National Inpatient Survey observed about 25,000 bariatric surgeries, with 3.2% mortality in the elderly compared with 0.2-0.7% in other age groups^34^. Nelson *et al*.^35^ analyzed 25 patients ≥ 65 years and showed 4% mortality and total complication rate in 20% of the cases. Pajecki *et al*.^24^ showed favorable results regarding weight loss and control of comorbidities in the elderly, and other studies showed adequate results of bariatric surgery in relation to weight loss and control of T2DM and hypertension in the elderly^19,28^.

## Conclusions

We conclude that laparoscopic RYGB is an effective method for controlling T2DM, DLP, and OSAS in obese elderly individuals (≥ 60 years) in the first 24 months of postoperative follow-up. In addition, it has been shown to be a safe procedure with low rates of minor complications that often do not require surgical intervention in this population. However, further studies with a larger sample of people involved are needed to ratify the results obtained in the present series.
